# Predicting Solubility Enhancement of *Trans*-Resveratrol and Hesperetin in Binary Solvent Mixtures Using New Hansen Parameters

**DOI:** 10.3390/molecules31071117

**Published:** 2026-03-28

**Authors:** Iván Montenegro, Ángeles Domínguez, Begoña González, Elena Gómez

**Affiliations:** FEQx Lab, Department of Chemical Engineering, University of Vigo, 36310 Vigo, Spain; ivan.montenegro@uvigo.gal (I.M.); admguez@uvigo.gal (Á.D.); bgp@uvigo.gal (B.G.)

**Keywords:** *trans*-resveratrol, hesperetin, solubility, Hansen solubility parameters, extraction, polyphenols

## Abstract

The solubility values of polyphenolic compounds in different extraction solvents are crucial for their recovery from natural matrices. Hansen solubility parameters (HSPs) stand out as a predictive tool for evaluating solute-solvent affinity and thus rational solvent selection for extraction processes. In this study, HSPs of *trans*-resveratrol and hesperetin were calculated using a semi-empirical method to assess the capability to predict the solubility behavior of both polyphenols in organic binary solvent mixtures. Experimental solubility of both polyphenols was determined in up to 21 monosolvents at 298.15 K and 0.1 MPa and used to classify them to iteratively calculate HSPs. Calculated HSPs were compared and discussed with literature values in terms of molecular interactions, demonstrating a fair agreement. Solubility of *trans*-resveratrol and hesperetin was then determined in methanol + MEK, ethanol + MEK, methanol + MiBK, ethanol + MiBK, and methanol + ethanol binary solvent mixtures. *trans*-Resveratrol achieved higher mole fraction solubility than hesperetin in all binary mixtures across the whole molar fraction range except in methanol + MiBK. Both compounds exhibited enhanced solubility in all alcohols + ketone binary mixtures, attributed to synergistic solvent effects. HSP analysis revealed a minimum Hansen distance between solute and solvent mixtures at compositions corresponding to the solubility maximum in synergistic systems. Additionally, calculated HSPs proved to effectively estimate the concentration at which this phenomenon occurs in all tested systems, reaching a robust correlation between maximum solubility and minimum Hansen distance. Overall, insights from this study underscore the effectiveness of experimentally derived HSPs in predicting the solubility behavior of polyphenols and seek to provide valuable guidance on solvent selection strategies for the recovery of bioactive compounds.

## 1. Introduction

Winemaking residues constitute an abundant and renewable source of natural antioxidants, which has led to increasing interest in their valorization for the recovery of high-added-value compounds [1,2]. Among the wide variety of compounds present in grape pomace, polyphenols stand out due to their remarkable antioxidant activity and their associated health-promoting properties. Within this group, *trans*-resveratrol and hesperetin are among the most abundant and industrially relevant polyphenols, owing to their biological activities and the increasing demand in several industrial sectors.

Hesperetin has been extensively studied due to its broad spectrum of bioactivities. Numerous experimental investigations have reported its strong antioxidant and anti-inflammatory properties, as well as its antitumor, cardioprotective, and neuroprotective effects [3,4]. These characteristics make hesperetin a compound of significant interest for applications in the pharmaceutical, nutraceutical, and functional food industries, where it is increasingly considered a natural bioactive ingredient.

*trans*-Resveratrol is one of the most intensively investigated polyphenols, particularly associated with grape skins and winemaking by-products. Its remarkable antioxidant capacity, together with cardioprotective, anti-inflammatory, anticancer, and neuroprotective activities [5,6], has supported its widespread use in dietary supplements, cosmetic formulations, and functional products.

The recovery of these compounds from natural sources strongly depends on the selection of an appropriate extraction solvent. Polyphenols generally exhibit low solubility in water, which limits the use of aqueous systems and requires the employment of organic solvents [7,8,9]. Consequently, methanol and ethanol, either as pure solvents or in hydro-alcoholic mixtures, have been traditionally used for the extraction of these compounds due to their relatively higher extraction yields, which have been reported to be up to 30% for polyphenols [10]. Despite their effectiveness, these solvents present limitations associated with toxicity, environmental impact and safety, which has motivated the search for alternative solvents within the context of green chemistry.

In this regard, the development of more environmentally friendly and sustainable extraction processes requires the investigation of the solubility behavior of target compounds in a wide range of solvents with improved environmental, health, and safety profiles. Knowledge of solubility data is essential not only to enhance extraction efficiency but also to design selective separation sequences that minimize solvent consumption and environmental impact. Moreover, it is well reported in the literature and has also been demonstrated in our previous work that binary solvent mixtures can exhibit a synergistic effect, leading to solubility values significantly higher than those observed in the corresponding pure solvents [11,12]. This phenomenon makes solvent mixtures particularly attractive for polyphenolic extraction and reinforces the importance of studying solubility not only in pure solvents but also in binary solvent systems.

The selection of suitable solvents for polyphenol extraction is increasingly guided by the principles of green chemistry, with the Safety, Health, and Environmental impact (SHE) framework serving as a standard evaluation tool [13]. This approach evaluates solvents based on three aspects: health, considering toxicity; safety, and environmental impact. Among the solvents classified as green, ketones such as methyl ethyl ketone (MEK) and methyl isobutyl ketone (MiBK) are notable due to their favorable SHE profiles [14]. For this reason, the binary solvent systems investigated in this study combined alcohols (methanol and ethanol) with ketones (MEK and MiBK), allowing the investigation of synergistic effects on polyphenol solubility while maintaining adherence to the principles of green chemistry.

Given the experimental work required to identify optimal solvent compositions, the ability to predict solubility maxima in solvent mixtures is highly desirable. Among the available theoretical approaches, Hansen solubility parameters (HSPs) have proven to be a very useful tool for the rational selection of solvents and prediction of solubility, as they describe the balance of dispersion, polar, and hydrogen-bonding interactions that govern solute–solvent affinity [15,16,17,18]. In addition to enabling the prediction of solubility trends and maxima in solvent mixtures, the experimental determination of HSPs for solid solutes remains particularly valuable, since such data are scarce and often estimated indirectly through group contribution methods [16] or predictive software [19,20]. In the case of polyphenolic compounds, experimentally determined HSP values are especially limited in the literature.

In one of our previous works, we determined the HSPs of quercetin and *p*-coumaric acid, two valuable polyphenols commonly found in winemaking by-products [21]. The results from that study revealed a robust correlation between the solubility enhancement of both polyphenols in binary solvent mixtures and the proximity degree in the Hansen space. Building on these findings, the present investigation focuses on *trans*-resveratrol and hesperetin, whose solubility was experimentally determined in 17 and 21 pure solvents at 298.15 K and 0.1 MPa, respectively, allowing for the calculation of their HSPs. In addition, solubility measurements were carried out in five binary solvent mixtures: methanol + MEK, ethanol + MEK, methanol + MiBK, ethanol + MiBK, and methanol + ethanol, in order to evaluate the synergistic effects of these mixtures. Based on the determined HSPs, the solubility maxima of *trans*-resveratrol and hesperetin in binary systems were predicted and subsequently assessed as a function of the functional groups present in the solvent mixtures. This work contributes to expanding the experimental solubility database of polyphenols, provides HSP values for these compounds, investigates the synergistic effect of binary solvent mixtures on solubility, and advances the rational design of greener and more efficient extraction processes.

## 2. Materials and Methods

### 2.1. Materials

**Figure 1:**
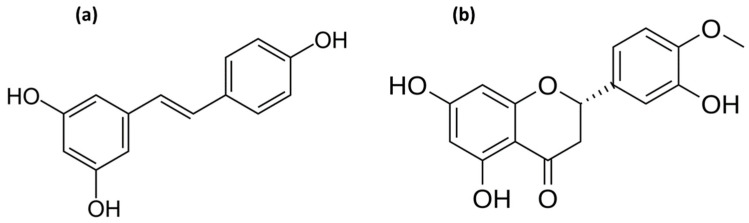
Structure of (**a**) *trans*-resveratrol and (**b**) hesperetin.

All chemicals used in this work were of analytical grade. Information regarding purity, supplier, CAS number, and purification procedures is provided in Table S1 (Supplementary Material). All solvents were used as received. Prior to experimentation, *trans*-resveratrol and hesperetin (chemical structures shown in Figure 1) were dried at 323.15 K for 3 h to remove residual moisture.

### 2.2. Methods

#### 2.2.1. Solubility Determination in Monosolvents

Solubility measurements of *trans*-resveratrol and hesperetin were conducted in a set of 17 and 21 monosolvents, respectively, at 298.15 K and 0.1 MPa in order to support the determination of their Hansen solubility parameters (Section 3.1). The solvent set was selected to ensure both chemical diversity and availability of reliable Hansen parameters. Accordingly, water; alcohols (methanol, ethanol, 1-propanol, and 1-octanol); ketones (acetone, methyl ethyl ketone (MEK), and methyl isobutyl ketone (MiBK)); acetonitrile; esters (ethyl acetate, *n*-propyl acetate, isopropyl acetate, *n*-butyl acetate, and ethyl lactate); alkanes (*n*-hexane and *n*-decane); cycloalkanes (cyclohexane and cyclooctane); diisopropyl ether; and *p*-xylene were investigated. Hansen solubility parameters for all solvents were taken from literature sources [15].

Solubility values of *trans*-resveratrol in methanol, ethanol, and ethyl acetate have been previously reported [22] and were therefore not re-determined in this study. For the remaining solvent systems, solubility was measured using an equilibrium-based method combined with UV–VIS spectroscopic analysis, following established procedures described elsewhere [23,24].

In brief, an excess amount of solute was added to 10 mL of solvent, and the suspension was stirred in a thermostated water bath at 298.15 K for 2 h. After equilibration, undissolved solids were removed via centrifugation (Hettich Universal 320, Sigma Aldrich, Tuttlingen, Germany) at 12,000 rpm for 20 min. Aliquots of the clear supernatant were withdrawn and diluted when necessary prior to analysis. Absorbance measurements were performed using a JASCO V-750 UV–VIS spectrophotometer (STA 449F3, Netzsch, Germany) at 305 nm for *trans*-resveratrol and 285 nm for hesperetin. Solubility values were calculated using external calibration curves prepared for each polyphenol in the corresponding solvents.

#### 2.2.2. Solubility Determination in Binary Solvent Mixtures

Based on the preliminary solubility results obtained in monosolvents, the mole fraction solubility of *trans*-resveratrol and hesperetin was evaluated at 298.15 K and 0.1 MPa over the entire mole fraction range. The investigated systems included methanol + MEK, ethanol + MEK, methanol + MiBK, ethanol + MiBK, and methanol + ethanol solvent mixtures.

For each experiment, an excess of solute was introduced into 0.5 mL of the prepared binary mixture contained in 1.5 mL Eppendorf tubes. Samples were equilibrated under continuous stirring in a thermostated water bath at 298.15 K for 2 h. Phase separation was achieved by centrifugation, after which a 0.1 mL aliquot of the saturated supernatant was collected, weighed, and allowed to evaporate to dryness. The resulting residue was re-dissolved in methanol and diluted as required to ensure absorbance values within the calibration range.

Solubility was determined by UV–VIS spectroscopy using calibration curves prepared in methanol at the maximum absorption wavelengths of each compound (305 nm for *trans*-resveratrol and 285 nm for hesperetin). Validation of this analytical protocol has been reported previously [22].

To ensure reliability of the experimental solubility measurements, solid-state characterization of *trans*-resveratrol and hesperetin was conducted via Powder X-Ray Diffraction (PXRD) prior to and after equilibrium with all solvents tested. Raw polyphenols and the remaining solid phases after centrifugation were introduced with an X-ray diffractometer (X’PERT PRO, PANalytical, Almelo, The Netherlands), and measurements were performed with a 40 kV voltage and 30 mA current, while the scanning angle was set from 2° ≤ 2θ ≥ 70°, and the step size was 0.026°. The results are displayed in Figures S1 and S2, and a brief analysis can be found in the Supplementary Information.

### 2.3. Hansen Solubility Parameters (HSPs)

#### 2.3.1. Theoretical Framework

The Hansen solubility parameter (HSP) framework describes the cohesive energy density of a compound as the sum of three intermolecular contributions: dispersion interactions (*E_D_*), polar interactions (*E_P_*), and hydrogen-bonding interactions (*E_H_*). When normalized by molar volume, these contributions define the total solubility parameter of the compound, as expressed in Equation (1) [15].

(1) $$\delta_{T}({\mathrm{MPa}}^{½})=\sqrt{\delta_{D}^{2}+\delta_{P\;}^{2}+\delta_{H}^{2}}$$

In Hansen space, solute–solvent affinity is evaluated through the Hansen distance (R_a_), defined as the Euclidean distance between the coordinates of the solute and solvent (Equation (2)).

(2) $$R_{a}=\sqrt{4\cdot {\left(\delta_{\mathrm{Da}}-\delta_{\mathrm{Db}}\right)}^{2}+{\left(\delta_{\mathrm{Pa}}-\delta_{\mathrm{Pb}}\right)}^{2}+{\left(\delta_{\mathrm{Ha}}-\delta_{\mathrm{Hb}}\right)}^{2}}$$

where the subscripts a and b refer to the solute and solvent, respectively. In this framework, a solvent that is highly miscible with the solute would be contained inside this sphere, while a solvent with low affinity with the solute would fall out of it. This compatibility is commonly assessed with the relative energy difference (RED), as expressed in Equation (3):

(3) $$\mathrm{RED}\;=\;R_{a}/R_{0}$$

In this regard, a suitable solvent would achieve a RED value lower than 1 for a given solute molecule, whilst values equal to or close to the unity would be considered boundary conditions, and progressively higher values would indicate lower affinity.

#### 2.3.2. Determination of HSPs

The Hansen solubility parameters of trans-resveratrol and hesperetin were determined experimentally based on their equilibrium solubility behavior in the selected set of pure solvents. Each solvent was classified as either “good” or “bad” according to the solubility criteria described in Section 3.1, providing the basis for the construction of the Hansen solubility sphere of each polyphenol.

Parameter calculation was carried out following the multi-response optimization approach proposed by Díaz & Hernández [25], implemented using the Solver tool in Microsoft Excel. Experimental solubility data obtained in this work were combined with Hansen solubility parameters of the solvents from literature [15] and introduced into the optimization worksheet provided by the authors (see the selection criteria in Section 3.1).

The optimization procedure aimed at determining the set of solubility parameters (δ_D_, δ_P_, δ_H_) and the interaction radius (R_0_) that best describe the experimental solubility behavior. To this end, an objective function known as the Size Factor was maximized. This function evaluates the quality of the fit by ensuring that all solvents classified as good are located within the solubility sphere, while bad solvents remain outside.

Initial estimates of the HSP values were automatically generated based on the average Hansen parameters of the good solvents. Constraints were applied to restrict the parameters to physically meaningful ranges, considering the chemical nature of polyphenolic compounds.

$$\left\{\delta_{D},\;\delta_{P},\;\delta_{H}\right\}\;\leq \;25\;;\;\delta_{D}\;\geq \;10\;;\;\left\{\delta_{P},\;\delta_{H}\right\}\;\geq \;5\;;\;10\;\geq \;R_{0\;}\geq \;1\;;\;R_{0\;}\geq \;R_{a,\;\mathrm{good},\;\mathrm{MAX}}$$

The first four are constraints of independent variables, and were set based on the guess values of HSP provided. The dispersion parameter is usually between 10 and 20 for non-alkane molecules, and polarity and hydrogen-bonding are higher than 5 for almost all polyphenols due to their structure and abundance of hydroxyl and carbonyl groups. On the other hand, all “good” solvents should be inside the sphere and “bad” ones should be outside, and the radius must be higher than the maximum distance between the polyphenol and all “good” solvents—that is to say, the greatest $R_{a}$ value attained for a “good” solvent ($R_{a,\;\mathrm{good},\;\mathrm{MAX}}$).

In addition, the sphere radius was constrained to exceed the maximum Hansen distance between the solute and any good solvent. Optimization was performed using the Evolutionary algorithm, as algorithm choice has been reported to have a negligible influence on the resulting HSP values [25].

More details about the multi-response optimization for calculating HSPs can be found in the work developed by Díaz & Hernández [25].

## 3. Results and Discussion

### 3.1. HSPs from Solubility Measurements in Monosolvents

In order to classify the solvents as “good” or “bad” for HSP calculation, the solubility of *trans*-resveratrol and hesperetin in 17 and 21 monosolvents, respectively, was experimentally determined at 298.15 K and 0.1 MPa. Results are collected in Table S2, along with values measured in previous works.

The solubility of both polyphenols could be quantified in all solvents except in *n*-hexane, *n*-decane, cyclohexane, cyclooctane, and *p*-xylene, since solute concentration could not be calibrated for being so insoluble in alkanes, cycloalcakanes and aromatic compounds. Consequently, stock solutions could not be prepared, and *n*-hexane, *n*-decane, cyclohexane, cyclooctane, and *p*-xylene were straightforwardly labeled as “bad” solvents for both polyphenols.

*trans-*Resveratrol exhibited a notorious solubilization in acetone, MEK, ethanol, and methanol, surpassing 100 g·L^−1^, while values descend below 34 g·L^−1^ in the rest of the solvents tested. As for hesperetin, experimental solubility did not exceed 40 g·L^−1^, except in acetone and MEK, where it was 102.80 g·L^−1^ and 77.66 g·L^−1^. There are three key commonalities in the solubility profile of both polyphenols: first, solubility decreases as the alkyl length of alcohols increases (methanol > ethanol > 1-propanol > 1-octanol); among all the esters tested, the highest solubility attained was achieved in ethyl lactate. Eventually, although both *trans*-resveratrol and hesperetin proved to be very soluble in acetone and MEK, solubility drops sharply in MiBK despite the carbonyl group, underscoring the unfavorable effect of simultaneously increasing the carbonic chain and introducing methyl branching.

In order to determine the HSPs of polyphenols, a criterion must be set for solvent classification. In this case, both *trans*-resveratrol and hesperetin achieved quite dispersed solubility values in the tested solvents, with almost equal relative standard deviations of 118% and 110% from average values, respectively, if alkanes, cycloalkanes, and *p*-xylene are excluded. As a first approach, the mean solubility was taken as a representative threshold for solvent discrimination, resulting in 38.84 g·L^−1^ for *trans*-resveratrol and 25.98 g·L^−1^ for hesperetin. However, both polyphenols attained slightly lower solubilities; for instance, the experimental solubility of hesperetin in *n*-propyl acetate is 18.62 g·L^−1^, which, despite being lower than the mean value, represents an appreciable degree of solubilization and thus might reasonably be considered a good solvent. Quite similar cases involve *trans*-resveratrol in 1-propanol, MiBK, ethyl lactate and ethyl acetate, with 33.38 g·L^−1^, 31.82 g·L^−1^, 27.14 g·L^−1^ and 20.89 g·L^−1^, respectively. Consequently, in view of the wide dispersion of solubility values and the multiple borderline cases, a threshold of 20 g·L^−1^ was adopted for solvent classification, considering that it represents a sufficiently high solubility to regard a solvent as “good”. This way, ethyl acetate was the lower limit for good solvents for *trans*-resveratrol, whereas an exception was made for hesperetin since solubility in *n*-propyl acetate achieved 18.62 g·L^−1^, a quite close value to the established limit. Eventually, ethanol, methanol, 1-propanol, acetone, MEK, MiBK, ethyl lactate, and ethyl acetate were labeled as “good” solvents for *trans*-resveratrol, whereas for hesperetin, this set included just methanol, acetone, MEK, MiBK, ethyl lactate, and *n*-propyl acetate.

Once the classification was performed, HSPs of *trans*-resveratrol and hesperetin were determined by performing several iterations within the previously described multi-response problem. Table 1 shows the results obtained, along with all HSPs found in the literature of the studied polyphenols. For comparative analysis, the relative deviation (RD) is defined in Equation (4).

(4) $$\mathrm{RD}=\left|\frac{\delta_{i}-\delta_{i,\mathrm{lit}}}{\delta_{i,\mathrm{lit}}}\right|100\;(\%)$$

where $\delta_{i}$ is the HSP calculated in this work, where $i\;=\;\left\{D,\;P,\;H,\;T\right\}$, and $\delta_{i,\mathrm{lit}}$ stands for the HSP taken from literature.

Iterative tests employing alternative solubility limits of 15 g·L^−1^ and 30 g·L^−1^ were conducted, showing negligible variations in the calculated HSPs. Considering the maximum solubilities observed, a threshold of 20 g·L^−1^ was therefore considered practically and methodologically appropriate.

HSPs of both polyphenols present a similar tendency regarding intermolecular interactions: the dispersion parameter, $\delta_{D}$, is almost equal to the hydrogen-bonding one, $\delta_{H}$, while a lower value was achieved for the polarity contribution $\delta_{P}$. As for the former partial HSP, hesperetin attained a slightly higher dispersion parameter than *trans*-resveratrol, with respective values of 12.7 MPa^½^ and 12.2 MPa^½^. Although both compounds contain two aromatic rings, hesperetin exhibits a bulkier conjugated system and less planar structure, which enhances dispersive interactions due to a larger effective molecular surface area, whilst *trans*-resveratrol presents a more planar and linear configuration. This latter also achieved a lower polarity parameter (9.9 MPa^½^) than hesperetin (10.3 MPa^½^) despite having the same number of hydroxyl groups attached. The slight difference might come from the presence of the extra carbonyl group of hesperetin, which induces a strong and well-defined dipole; eventually, hesperetin attains a higher global polarity than *trans*-resveratrol, which also matches with log *p* values available elsewhere [32]. Regarding hydrogen-bonding contribution, results revealed similar values for both polyphenols, with 12.7 MPa^½^ for *trans*-resveratrol and 12.5 MPa^½^ for hesperetin, which seems coherent considering that both have three hydrogen bond donor groups. Although hesperetin has a stronger acceptor character due to the carbonyl group and methoxy oxygen, while *trans*-resveratrol presents only three weak hydrogen bond acceptors, the latter achieved a slightly higher partial HSP. This could be attributed to the greater accessibility of the hydroxyl groups of resveratrol, which is favored by its less sterically hindered structure, whereas the flavonoid scaffold of hesperetin may, to some extent, limit its hydrogen-bonding capability [33].

As far as we are concerned, this is the first work to report HSPs of *trans*-resveratrol and hesperetin calculated using this approach. Nevertheless, in order to perform a literature comparison, HSPs of both polyphenols determined by other methods are shown in Table 1. Kanda et al. [28], Ghazwani et al. [27], and Yu et al. [26] obtained the HSPs of *trans*-resveratrol using HSPiP software (v4.1.07, Louisville, KY, USA), while Shi et al. [29] employed the group contribution (GC) method of Beerbower to calculate them. These HSPs from the literature follow the same trend of our results regarding the polarity contribution, being the lowest value reported by Kanda et al. [28], Ghazwani et al. [27], and Shi et al. [29], whereas only the total HSP is available in the work developed by Yu et al. [26]. The most notable discrepancy with our results is observed in the dispersion parameter, with RDs of approximately 70% with respect to $\delta_{D}$ of Kanda et al. [28], Ghazwani et al. [27], and Shi et al. [29]. However, using Díaz & Hernández’s [25] methodology, the dispersion contribution would unlikely attain values comparable to those calculated by structure-based methods. This is consistent with the experimental observation that polyphenols are highly insoluble in solvents with large dispersion parameters, such as alicyclic hydrocarbons or long-chain alkanes. Consequently, iterative approaches based on experimental solubility data inhibit excessive dispersive interactions and lead to lower δ_D_ values. In contrast, SMILES-based or GC methods may overestimate London forces by solely relying on molecular structure. As for polarity, better comparability agreement was achieved, with RDs ranging from 26. 3% to 49.5%. Nonetheless, discrepancies of up to 31.5% are also observed among literature values themselves, particularly between Shi et al. [29] and Ghazwani et al. [27], underscoring the strong influence of the selected methodology on the resulting HSPs. The hydrogen-bonding parameter of *trans*-resveratrol obtained in the present work is fully consistent with that obtained by Kanda et al. [28] and Shi et al. [29], yielding RDs of 3.1% and 2.3%, respectively. In contrast, a markedly larger discordance is observed with the value reported by Ghazwani et al. [27], deviating by 25.2%. For its part, Yu et al. [26] only reported a total HSP of 25.51 MPa^½^ of *trans*-resveratrol, which deviates by 26.3% from our value of 20.2 MPa^½^, although the absence of partial parameters precludes a more detailed comparison and further discussion.

With regard to hesperetin, only two sets of HSPs were found in the literature, both calculated by means of the GC method developed by Van Krevelen-Hoftyzer. The difference between these studies lies in the calculation of the molecular volume of hesperetin, for which Berga et al. [31] used HSPiP software (v5.1.03, Louisville, KY, USA), while Han et al. [30] proceeded as originally outlined in the method. The trend observed in the HSPs from both studies agrees with ours in terms of the difference between the polarity parameter and the other two, as also happens with *trans*-resveratrol. HSPs reported by Berga et al. [31] achieve RDs of 61.4%, 49.5%, and 38.5% compared to our dispersion, polarity, and hydrogen-bonding parameters, respectively; in contrast, overall better accordance is revealed with values calculated by Han et al. [30], with respective RDs of 39.3%, 2.9%, and 34.4%. In both cases, the highest deviation corresponds to the dispersion parameter, as observed for *trans*-resveratrol. In addition, the polarity parameter obtained by Berga et al. [31] is slightly higher than half of the values reported by Han et al. [30] and that obtained in this work. These insights outline the discordances encountered when selecting one method or another to determine HSPs.

Overall, HSPs of polyphenols found in literature appear, to a certain extent, inconsistent with the intermolecular behavior of the molecules. Despite *trans*-resveratrol standing out for its hydrogen-bonding capability, all reported dispersion parameters largely exceed the hydrogen-bonding ones, which does not reflect the dominant interactions that govern solubility behavior, according to experimental results obtained in this investigation. Furthermore, even if a literature comparison is performed as a function of the polyphenol considered rather than by individual HSPs, the results still do not align with the expected behavior of the two compounds. In particular, although hesperetin could be expected to show a higher dispersion parameter than *trans*-resveratrol due to structural and size differences, the reported HSP values reflect an opposite behavior across all different combinations of data taken from the literature, regardless of the calculation approaches employed. This observation may suggest inherent limitations in directly comparing HSPs derived using different methodologies.

**Figure 2:**
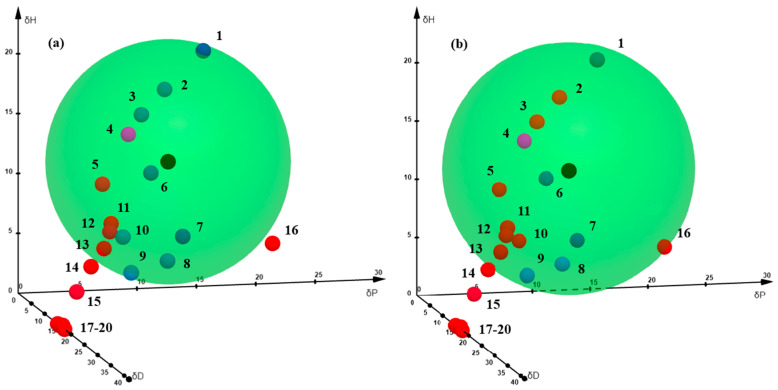
Hansen space of (**a**) *trans*-resvetratrol and (**b**) hesperetin. HSPs of polyphenols; “good” and “bad” solvents are represented with black, blue and red dots, respectively. Hansen spheres are shown in green, and pink dots refer to HSPs of 1-butanol. Numbered solvents include methanol (1), ethanol (2), 1-propanol (3), 1-butanol (4), 1-octanol (5), ethyl lactate (6), acetone (7), MEK (8), MiBK (9), ethyl acetate (10), isopropyl acetate (11), *n*-propyl acetate (12), *n*-butyl acetate (13), diisopropyl ether (14), p-xylene (15), acetonitrile (16), alkanes and cycloalkanes (17–20). This 3D representation of the Hansen space is intended for visual interpretation only; thus, distances between points do not correspond to Ra values used in HSP calculations and should not be interpreted quantitatively according to Hansen theory.

Figure 2 displays the Hansen spheres obtained from the multi-response program optimization. An 80% data fit was reached for *trans*-resveratrol, whereas hesperetin achieved a 75%, with all solvents labeled as “good” correctly located inside the respective Hansen spheres in both cases. Even though several solvents are contained inside both spheres despite being categorized as “bad”, only two and four of them actually exhibited RED values below 1 compared to *trans*-resveratrol and hesperetin HSPs, respectively. This discrepancy arises because a distance calculated for a three-dimensional representation does not include the factor 4 (see Section 2.3.1), which is indeed used in Equation (2) to calculate the Hansen distances for HSP determination of both polyphenols. Consequently, since HSPs are employed as a tool for solvent selection, the distances must be calculated as mentioned; RED values should take precedence over the distances observed in Figure 2 [15], which is provided solely for visual reference. Isopropyl acetate and *n*-propyl acetate are the two “bad solvents” contained in the Hansen sphere of *trans*-resveratrol, with RED values of 0.88 and 0.97, respectively. These anomalies stem from the inherent sensitivity of the Hansen theory near the boundaries of the sphere [15], since both RED values are quite close to unity. However, more inconsistencies were identified in the hesperetin sphere, concerning ethanol, 1-propanol, ethyl acetate, and isopropyl acetate, all with REDs ranging from 0.80 to 0.90, underscoring close proximity to the edges of the hesperetin sphere.

All in all, it can be inferred that the group of solvents that caused more anomalies in the Hansen space for both polyphenols was acetates, while hesperetin presented extra inconsistencies with two alcohols. This could be explained by the contrast between the expected behavior of the molecule and the actual experimental solubility values obtained. Although hesperetin contains one ketone group and multiple hydroxyl groups attached, its solubility in primary alcohols is quite low, with the exception of methanol. This highlights the importance of supporting computationally derived results with experimental solubility data to ensure reliability.

Eventually, experimental solubility of *trans*-resveratrol and hesperetin was determined in 1-butanol to test the effectiveness of the Hansen spheres obtained; thus, HSPs of this solvent are also displayed in Figure 2. According to Hansen theory, 1-butanol should be considered as a good solvent for both polyphenols, with RED values of 0.91 and 0.82 for *trans*-resveratrol and hesperetin, respectively. Regarding *trans*-resveratrol, experimental solubility in 1-butanol resulted in 18.38 g·L^−1^, a very proximate value to the threshold previously set to 20 g·L^−1^, suggesting boundary conditions that agree with the high RED value achieved. As for hesperetin, an experimental solubility value of 7.71 g·L^−1^ in 1-butanol clearly rules it out as a good solvent despite falling inside the Hansen sphere. Nonetheless, 1-butanol follows the trend identified in ethanol and 1-propanol, which were considered boundary cases for hesperetin with RED values close to unity. Hence, having analyzed the correlation between experimental solubility values of hesperetin in these two alcohols and knowing their location in the Hansen space, 1-butanol would have been initially considered a bad solvent, even in the absence of any experimental testing.

### 3.2. Binary Solvent Mixtures

#### 3.2.1. Experimental Solubility

**Figure 3:**
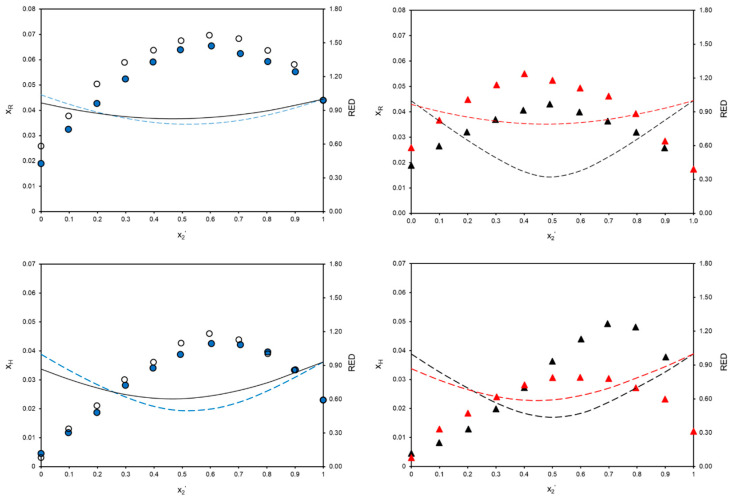
Mole fraction solubility of *trans*-resveratrol (x_R_) and hesperetin (x_H_) in methanol + MEK (●), ethanol + MEK (◯), methanol + MiBK (▲), and ethanol + MiBK (▲), as a function of the mole fraction of ketone solvent in the binary solvent mixture (x_2_’), at 298.15 K and 0.1 MPa. Lines represent the RED value between the R_a_ of each binary solvent mixture with respect to both polyphenols and R_0_, as a function of x_2_’: methanol + MEK (– – –), ethanol + MEK (––), methanol + MiBK (– – –), and ethanol + MiBK (– – –).

The solubility of *trans*-resveratrol and hesperetin was experimentally determined in methanol + MEK, ethanol + MEK, methanol + MiBK, ethanol + MiBK, and methanol + ethanol binary solvent mixtures at 298.15 K and 0.1 MPa. Although acetone yielded higher solubility values than the other ketone solvents for both polyphenols, MEK and MiBK were selected due to their more favorable SHE indicators and their potential to provide new solubility data, thus expanding the current solubility database of *trans*-resveratrol and hesperetin. Solubility profiles of both polyphenols in the binary system composed of both alcohols are plotted in Figure S3 in the Supplementary information, while the others are plotted in Figure 3. Experimental solubility values can be consulted in Table S3 in the Supplementary Information. No literature comparison could be performed due to the absence of experimental solubility data available.

Figure 3 reveals solubility enhancement of *trans*-resveratrol and hesperetin in all alcohol + ketone solvent mixtures compared to solubility in monosolvents as a result of the synergistic behavior between the two components of the binary solvent system. Both polyphenols showed higher mole fraction solubility in ethanol + MEK than in methanol + MEK across the entire composition range. However, as far as MiBK is concerned, mole fraction solubility of *trans*-resveratrol in ethanol + MiBK remained above that in methanol + MiBK through the molar fraction range, while as for hesperetin, the latter surpassed the former from a MiBK mole fraction of 0.5 onward.

*trans*-Resveratrol achieved higher maximum mole fraction solubility values than hesperetin in methanol + MEK, ethanol + MEK, and ethanol + MiBK, whereas in methanol + MiBK, the synergistic effect between the solvents preferentially favored hesperetin solvation, particularly at high ketone concentrations. With regard to the concentration at which this phenomenon occurs, both *trans*-resveratrol and hesperetin achieved the solubility maximum at a 0.6 mole fraction of ketone solvent in methanol + MEK and ethanol + MEK binary solvent mixtures, respectively. The maximum solubility of *trans*-resveratrol in methanol + MiBK corresponds to a 0.5 mole fraction of MiBK, whereas in ethanol + MiBK, it is achieved at a 0.4 mole fraction of MiBK. As for hesperetin, the greatest enhancement occurs at higher ketone concentrations, with values of 0.7 and 0.6 mole fractions of MiBK in methanol + MiBK and ethanol + MiBK binary solvent mixtures, respectively.

As observed, *trans*-resveratrol and hesperetin showed enhanced solubility in all binary mixtures tested, composed of solvents with different functional groups, i.e., alcohol and ketone. In order to study the opposite case, the experimental mole fraction solubility of both polyphenols was determined in a methanol + ethanol binary solvent mixture, and the results are plotted in Figure S3. The solubility profiles exhibited a linear dependence of solubility on the concentration of the binary solvent mixtures for both polyphenols, indicating the absence of any synergistic effects among alcohols in the tested systems, thereby highlighting the critical role of functional group combinations in the solubility enhancement of polyphenols.

#### 3.2.2. HSPs for Solubility Prediction in Binary Solvent Mixtures

Experimental solubility profiles evidenced the enhanced mole fraction solubility of *trans*-resveratrol and hesperetin in all tested binary solvent mixtures composed of solvents with different functional groups, whereas solubility behaved linearly in the mixture formed by methanol and ethanol. According to the basis of the Hansen theory, the lower the RED between one solute and a solvent (or solvent mixture), the higher the affinity among them. Consequently, a solubility maximum should match a minimum RED value. To assess the predictability of this proposed correlation, RED values between both polyphenols and all binary solvent mixtures must be calculated across the entire molar fraction range. For this purpose, Equation (5) [34] was used to obtain the HSPs of the binary mixtures.

(5) $$\delta_{i}({\mathrm{MPa}}^{½})=\delta_{1}x_{1}+{\;\delta }_{2}(1-x_{1})$$

where $\delta_{i}$ represents the HSP of the binary solvent mixture with a mole fraction $x_{1}$ of solvent 1, where $i\;=\;\left\{D,\;P,\;H\right\}$ and $x_{1}$ varying from 0.1 to 0.9 in steps of 0.1. Parameters $\delta_{1}$ and $\delta_{2}$ correspond to the HSPs of monosolvents 1 and 2, respectively.

R_a_ distances between the HSPs of each polyphenol and binary solvent mixtures were calculated with Equation (2), covering the whole molar fraction range. Subsequently, RED values were determined with R_0_ of *trans*-resveratrol and hesperetin according to Equation (3), and the results are displayed in Figure 3.

As can be observed, all RED profiles plotted in Figure 3 show a minimum value at a given composition of the binary solvent mixture, with different degrees of curvature depending on the case. Regarding *trans*-resveratrol, minimum REDs are remarkably less pronounced in solvent mixtures containing MEK as well as in ethanol + MiBK than in the other binary solvents tested, suggesting a weaker dependence of solute–solvent interactions on the solvent mixture composition. Conversely, the RED trends of hesperetin in all alcohol + ketone systems tested and *trans*-resveratrol in methanol + MiBK exhibited distinct convex behavior, with a clear minimum RED value at a specific concentration of the binary solvent mixture.

Furthermore, the mole fraction of ketone solvent at which this minimum RED seems to fairly correspond to the composition that yielded maximum solubility enhancement in all alcohol + ketone systems. For instance, *trans*-resveratrol reaches maximum solubility and minimum RED at 0.6 and 0.5 mole fractions of MEK in alcoholic binary mixtures, respectively, which entails a close correspondence despite the flatness of RED profiles. Similarly, maximum enhancement of *trans*-resveratrol solubility and minimum Hansen distance in ethanol + MiBK are attained at 0.4 and 0.5 mole fraction of MiBK, whilst in methanol + MiBK, both phenomena coincide at a 0.5 mole fraction of the ketone in the binary solvent mixture. With regard to hesperetin, the correlation between solubility maxima and minimum RED in methanol + MEK and ethanol + MEK coincides with that of *trans*-resveratrol for these binary mixtures. On the other hand, maximum solubility enhancement and minimum Hansen distance of hesperetin in binary solvents containing MiBK occurred at 0.7 and 0.5 mole fraction of ketone solvent in methanol + MiBK and at 0.6 and 0.4 in ethanol + MiBK, respectively.

This correspondence was also checked for *trans*-resveratrol and hesperetin in a methanol + ethanol binary solvent mixture, which exhibited no solubility maximum in either of both cases. Figure S3 displays the RED trends of both ternary systems, from which it can be inferred that no minimum value was reached across the entire molar fraction range. As a result, HSP accurately predicted the solubility behavior of these systems, anticipating a non-synergistic behavior among solvents.

This strong agreement between experimental results and theoretical predictions indicates that the calculated HSPs can be effectively used to assess intermolecular interactions among solvents in binary mixtures to determine if solubility enhancement phenomena of *trans*-resveratrol and hesperetin are expected or not, as it was already reported for *p*-coumaric acid and quercetin [21]. Moreover, these findings enable accurate estimation of the solvent composition corresponding to solubility maxima of the aforementioned polyphenols.

## 4. Conclusions

Hansen solubility parameters of *trans*-resveratrol and hesperetin were derived from experimental solubility data, with the aim of assessing their capability to predict the solubility behavior of both polyphenols in binary solvent mixtures. Experimental solubility of *trans*-resveratrol and hesperetin was determined in up to 21 solvents with different functional groups, at 298.15 K and 0.1 MPa, in order to classify them following the method proposed by Díaz & Hernández [25] for HSP calculation. Hansen spheres showed good consistency with the experimental results and molecular behavior of polyphenols, reinforcing the reliability of the methodology applied and highlighting the importance of integrating experimental data with theoretical approaches. Calculated HSPs were compared to those available in the literature, attaining a certain degree of agreement, mainly attributed to several inconsistencies found between partial HSPs of referenced values and the expected molecular behavior of the studied compounds.

Following previous evidence of solubility enhancement of polyphenols in organic binary solvent mixtures, the mole fraction solubility of *trans*-resveratrol and hesperetin at 298.15 K and 0.1 MPa was determined in several binary solvent mixtures containing methanol, ethanol, MEK, and MiBK. Both polyphenols exhibited solubility enhancement in all alcohol + ketone binary mixtures tested due to the synergistic effect of solvents with different functional groups, with *trans*-resveratrol surpassing hesperetin in maximum mole fraction solubility achieved except in methanol + MiBK. Conversely, solubility maxima were not observed in ternary systems containing both alcohols.

The performance of obtained HSPs to predict solubility behavior of *trans*-resveratrol and hesperetin in the abovementioned binary solvents mixtures was evaluated by calculating the RED profiles between both polyphenols and the binary systems over the entire molar fraction range. *trans*-resveratrol and hesperetin achieved a minimum RED value in all alcohol + ketone binary mixtures, whereas a linear trend was revealed in methanol + ethanol for both polyphenols. Additionally, the composition at which the minimum RED is reached proved to closely match that at which maximum solubility enhancement occurs. These insights demonstrate that the semi-empirically derived HSPs are capable not only of anticipating solubility enhancement of polyphenols in organic binary mixtures but also of estimating the composition corresponding to the solubility maximum.

This evidence obtained for *trans*-resveratrol and hesperetin further complements previous findings reported for quercetin and *p*-coumaric acid [21], serving to emphasize the importance of combining experimental and computational approaches to the rational selection of solvents for polyphenol recovery.

## Figures and Tables

**Table 1:** HSPs ($\delta_{i}$), sphere radius ($R_{0}$) and data fit results of *trans*-resveratrol and hesperetin obtained in this work with the method reported by Díaz & Hernández [25]. All HSPs of both compounds found in literature are included for comparison and discussion.

Polyphenol	$\delta_{D}/{\mathrm{MPa}}^{½}$	$\delta_{P}/{\mathrm{MPa}}^{½}$	$\delta_{H}/{\mathrm{MPa}}^{½}$	$\delta_{T}/{\mathrm{MPa}}^{½}$	$R_{0}/{\mathrm{MPa}}^{½}$	Data Fit/%
*trans*-Resveratrol (this work)	12.2	9.9	12.7	20.2	10.1	80
*trans*-Resveratrol [26]	-	-	-	25.51	-	-
*trans*-Resveratrol [27]	20.60	7.30	15.90	27.10	-	-
*trans*-Resveratrol [28]	20.9	6.7	13.1	25.56	-	-
*trans*-Resveratrol [29]	21.0	5.0	13.0	25.20	-	-
Hesperetin (this work)	12.7	10.3	12.5	20.58	10.7	75
Hesperetin [30]	17.69	10.60	17.31	26.92	-	-
Hesperetin [31]	20.50	5.20	16.80	27.0	-	-

## Data Availability

The original contributions presented in this study are included in the article/Supplementary Material. Further inquiries can be directed to the corresponding author.

## Supplementary Materials

The following supporting information can be downloaded at: https://www.mdpi.com/article/10.3390/molecules31071117/s1, Table S1: Information of chemicals used for experimentation. Table S2: Experimental solubility of *trans*-resveratrol and hesperetin in pure solvents, measured at 298.15 K and 0.1 MPa. Table S3: Experimental mole fraction solubility of *trans*-resveratrol and hesperetin in binary solvent mixtures, measured at 298.15 K and 0.1 MPa. Figure S1: Powder X-ray diffraction profiles of *trans*-resveratrol after solubilization in monosolvents. Figure S2: Powder X-ray diffraction profiles of hesperetin after solubilization in monosolvents. Figure S3: Experimental mole fraction solubility of *trans*-resveratrol and hesperetin in methanol + ethanol binary solvent mixture, measured at 298.15 K and 0.1 MPa. Reference [22] is cited in the Supplementary Materials.
