# Supplementary material for: Predicting Solubility Enhancement of Trans-Resveratrol and Hesperetin in Binary Solvent Mixtures Using New Hansen Parameters
Source: Molecules. 2026 Mar 28;31(7):1117. doi: 10.3390/molecules31071117 (PMC13074312; doi:10.3390/molecules31071117)
Supplement: Supplementary file 1 [file molecules-31-01117-s001.zip › molecules-4115130-supplementary.pdf]

Predicting Solubility Enhancement of *trans*-Resveratrol and Hesperetin in Binary Solvent Mixtures Using New Hansen Parameters

Iván Montenegro, Ángeles Domínguez, Begoña González and Elena Gómez *

FEQx Lab, Department of Chemical Engineering, University of Vigo, 36310 Vigo, Spain;
ivan.montenegro@uvigo.gal (I.M.); admguez@uvigo.gal (Á.D.); bgp@uvigo.gal (B.G.)
* Correspondence: elenagc@uvigo.es

Supplementary material

- Table S1 Information of chemicals used for experimentation.
- Table S2 Experimental solubility of *trans*-resveratrol and hesperetin in pure solvents, measured at 298.15 K and 0.1 MPa.
- Table S3 Experimental mole fraction solubility of *trans*-resveratrol and hesperetin in binary solvent mixtures, measured at 298.15 K and 0.1 MPa.
- Figure S1 Powder X-ray diffraction profiles of (a) raw *trans*-resveratrol and solid phase after solubilization in (b) ethanol, (c) 1-propanol, (d) 1-butanol, (e) 1-octanol, (f) water, (g) acetone, (h) MEK, (i) MiBK, (j) acetonitrile, (k) isopropyl acetate, (l) diisopropil ether, (m) ethyl lactate.
- Figure S2 Powder X-ray diffraction profiles of (a) raw hesperetin and solid phase after solubilization in (b) methanol, (c) ethanol, (d) 1-propanol, (e) 1-butanol, (f) 1-octanol, (g) acetone, (h) MEK, (i) MiBK, (j) acetonitrile, (k) ethyl acetate, (l) isopropyl acetate, (m) *n*-propyl acetate, (n) *n*-butyl acetate, (o) ethyl lactate (p) diisopropyl ether.
- Figure S3 Experimental mole fraction solubility of *trans*-resveratrol and hesperetin in methanol + ethanol binary solvent mixture, measured at 298.15 K and 0.1 MPa.

Table S1. Information of chemicals used for experimentation

Compound	CAS number	Source	Molar mass/ $g \cdot mol^{-1}$	Mass purity[a]/%	Purification method
trans-Resveratrol	501-36-0	TCI	228.25	> 99.0	Drying[b]
Hesperetin	520-33-2	TCI	302.28	> 97.0	Drying[b]
Water	7732-18-5	CACTI facilities	18.02	MILLI-Q quality	NA[c]
Methanol	67-56-1	Sigma-Aldrich	32.04	≥ 99.9	NA[c]
Ethanol	64-17-5	Sigma-Aldrich	46.07	≥ 99.5	NA[c]
1-Propanol	71-23-8	VWR	60.09	≥ 99.7	NA[c]
1-Butanol	71-36-3	Sigma-Aldrich	74.12	99.9	NA[c]
1-Octanol	111-87-5	ACROS Organic	130.23	≥ 99	NA[c]
Acetone	67-64-1	Sigma Aldrich	58.08	≥ 99.8	NA[c]
MEK	78-93-3	Thermo Scientific	72.11	≥ 99	NA[c]
MiBK	108-10-1	Sigma-Aldrich	100.10	≥ 99.5	NA[c]
Acetonitrile	75-05-8	Fisher Chemical	41.05	≥ 99.9	NA[c]
Ethyl acetate	141-78-6	Sigma-Aldrich	88.11	≥ 99.7	NA[c]
n-Propyl acetate	109-60-4	Sigma-Aldrich	102.13	≥ 99	NA[c]
Isopropyl acetate	108-21-4	Sigma Aldrich	102.13	≥ 99.6	NA[c]
n-Butyl acetate	123-86-4	Sigma-Aldrich	116.16	≥ 99.5	NA[c]
Ethyl lactate	97-64-3	Sigma Aldrich	118.13	≥ 98	NA[c]
n-Hexane	110-54-53	Scharlau	86.18	99	NA[c]
n-Decane	124-18-5	Merck	142.29	≥ 99	NA[c]
Cyclohexane	110-82-7	Fisher Chemical	84.16	≥ 99.96	NA[c]
Cyclooctane	292-64-8	Sigma Aldrich	112.21	≥ 99	NA[c]
Diisopropyl ether	108-20-3	Sigma Aldrich	102.17	98.5	NA[c]
p-Xylene	106-42-3	Sigma Aldrich	106.16	≥ 99.0	NA[c]

[a] Provided by the supplier

[b] At 323.25 K for three hours

[c] Not applicable

Table S2. Experimental solubility of *trans*-resveratrol (S_R) and hesperetin (S_H) in pure solvents at 298.15 K and 0.1 MPa.

Solvent	$S_R \pm SD^1/g\cdot L^{-1}$	$S_H \pm SD^1/g\cdot L^{-1}$	Solvent	$S_R \pm SD^1/g\cdot L^{-1}$	$S_H \pm SD^1/g\cdot L^{-1}$
Water	0.06 ± 0.01	BQL[2]	*n*-Propyl acetate	11.85 ± 0.99 [1]	18.61 ± 0.53
Methanol	106.8 ± 2.82 [1]	33.54 ± 0.72	Isopropyl acetate	4.28 ± 0.29	10.36 ± 0.67
Ethanol	100.98 ± 1.96	16.05 ± 0.26	*n*-Butyl acetate	8.042 ± 0.87 [1]	8.72 ± 0.43
1-Propanol	33.38 ± 0.66	14.77 ± 0.60	Ethyl lactate	27.14 ± 0.68	39.08 ± 1.28
1-Butanol	18.38 ± 0.29	7.71 ± 0.41	*n*-Hexane	BQL[2]	BQL[2]
1-Octanol	6.97 ± 0.06	6.20 ± 0.07	*n*-Decane	BQL[2]	BQL[2]
Acetone	133.03 ± 1.37	102.80 ± 5.78	Cyclohexane	BQL[2]	BQL[2]
MEK	111.34 ± 1.27	77.66 ± 8.77	Cyclooctane	BQL[2]	BQL[2]
MiBK	31.82 ± 0.91	29.29 ± 1.07	Diisopropyl ether	0.35 ± 0.01	0.81 ± 0.04
Acetonitrile	5.63 ± 0.04	13.50 ± 0.15	*p*-Xylene	BQL[2]	BQL[2]
Ethyl acetate	20.89 ± 1.17 [1]	10.62 ± 0.63			

[1] Standard deviation (n = 3)

[2] Below quantification limit

Table S3. Experimental mole fraction solubility of *trans*-resveratrol (x_R) and hesperetin (x_H) in methanol + MEK, ethanol + MEK, methanol + MiBK, and ethanol + MiBK binary solvent mixtures at 298.15 *K* and 0.1 MPa, all expressed as a function of the mole fraction of the ketone solvent (x_{MEK}, x_{MiBK}) in the alcohol + ketone binary mixture and of ethanol (x_{ET}) in methanol + ethanol binary mixture[a].

Methanol + MEK			
x_{MEK}	x_R	x_{MEK}	x_H
0.0000	0.0189	0.0000	0.0045
0.0976	0.0325	0.0976	0.0117
0.1979	0.0427	0.1979	0.0187
0.2990	0.0524	0.2990	0.0281
0.3967	0.0590	0.3967	0.0341
0.4942	0.0639	0.4942	0.0387
0.6037[1]	0.0654	0.6037[1]	0.0425
0.7061	0.0624	0.7061	0.0421
0.8034	0.0593	0.8034	0.0396

0.9014	0.0552	0.9014	0.0334
1.0000	0.0439	1.0000	0.0230
Ethanol + MEK			
x_{MEK}	x_R	x_{MEK}	x_H
0.0000	0.0258	0.0000	0.0031
0.0982	0.0378	0.0982	0.0130
0.1982	0.0504	0.1982	0.0210
0.2958	0.0589	0.2958	0.0301
0.3985	0.0637	0.3985	0.0361
0.4965	0.0675	0.4965	0.0427
0.5962[1]	0.0696	0.5962[1]	0.0459
0.7005	0.0683	0.7005	0.0438
0.8035	0.0636	0.8035	0.0390
0.8969	0.0580	0.8969	0.0333
1.0000	0.0439	1.0000	0.0230
Methanol + MiBK			
x_{MiBK}	x_R	x_{MiBK}	x_H
0.0000	0.0189	0.0000	0.0045
0.0983	0.0265	0.0989	0.0082
0.1974	0.0320	0.2023	0.0129
0.2981	0.0370	0.3003	0.0198
0.3968	0.0406	0.4003	0.0272
0.4913[1]	0.0430	0.4995	0.0363
0.5960	0.0399	0.6016	0.0440
0.6967	0.0363	0.6951[1]	0.0493
0.7976	0.0320	0.7949	0.0481
0.8974	0.0258	0.9016	0.0378
1.0000	0.0174	1.0000	0.0121
Ethanol + MiBK			

x_{MiBK}	x_R	x_{MiBK}	x_H
0.0000	0.0258	0.0000	0.0031
0.0992	0.0367	0.0992	0.0129
0.2003	0.0448	0.2003	0.0184
0.3025	0.0507	0.3025	0.0240
0.4013[1]	0.0550	0.4013	0.0282
0.5004	0.0524	0.5004	0.0307
0.5987	0.0493	0.5987[1]	0.0308
0.6998	0.0462	0.6998	0.0303
0.7954	0.0393	0.7954	0.0271
0.8996	0.0285	0.8996	0.0233
1.0000	0.0174	1.0000	0.0121
Methanol + Ethanol			
x_{ET}	x_R	x_{ET}	x_H
0.0000	0.0275	0.0000	0.0045
0.0976	0.0309	0.0976	0.0043
0.1979	0.0317	0.1979	0.0043
0.2990	0.0322	0.2990	0.0042
0.3967	0.0330	0.3967	0.0040
0.4942	0.0331	0.4942	0.0041
0.6037	0.0333	0.6037	0.0040
0.7061	0.0334	0.7061	0.0040
0.8034	0.0334	0.8034	0.0038
0.9014	0.0335	0.9014	0.0035
1.0000	0.0335	1.0000	0.0031

[a]The standard uncertainty of temperature is u(T) = 0.1 K, and that of pressure is u(P) = 1 kPa. Standard uncertainty for all mole fraction solubility values is $u(x_q\ x_c) = 0.0005$, and that for the mole fraction of secondary solvent in the binary mixture is $u(x_{MEK}, x_{MiBK}, x_{ET}) = 0.0002$ [1].

[1]Mole fraction of ketone solvent in the binary solvent mixture at which maximum solubility is achieved.

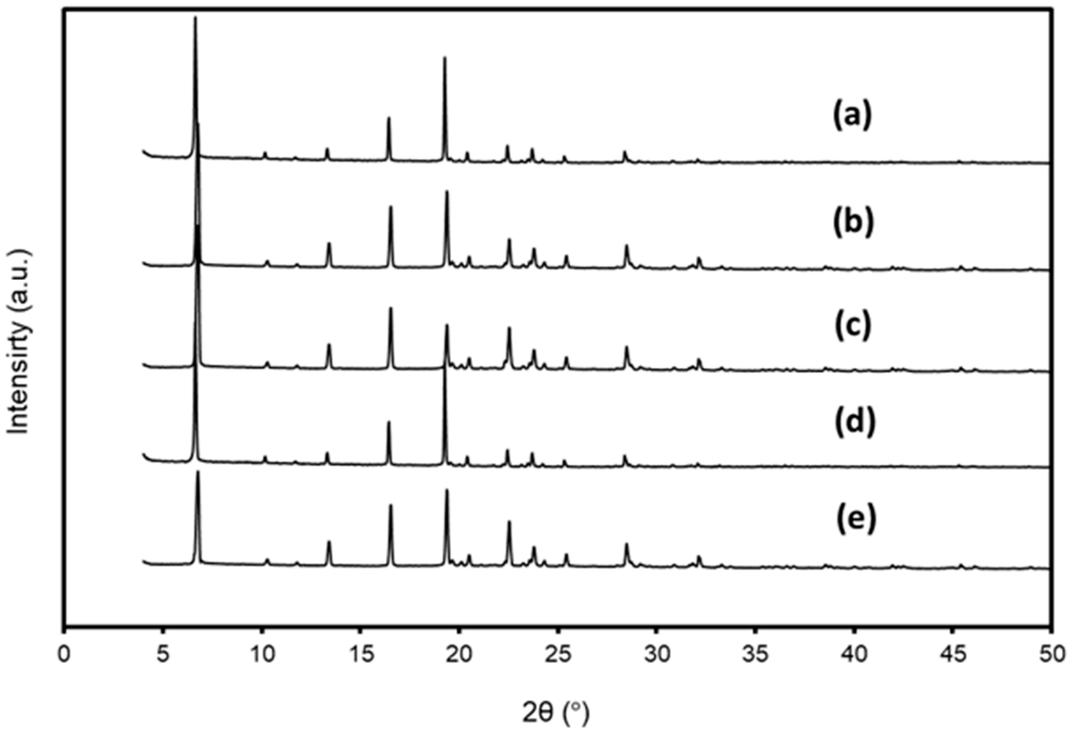

Intensirty (a.u.)
(a)
(b)
(c)
(d)
(e)
0
5
10
15
20
25
30
35
40
45
50
2θ (°)

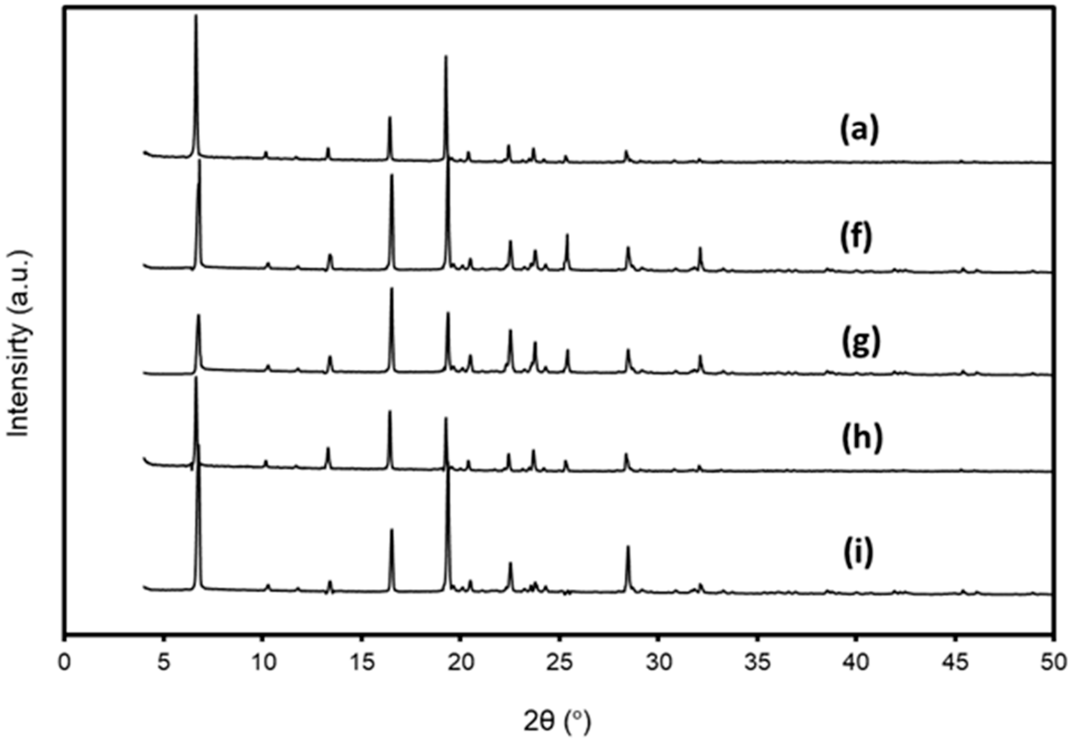

Intensirty (a.u.)
(a)
(f)
(g)
(h)
(i)
0
5
10
15
20
25
30
35
40
45
50
2θ (°)

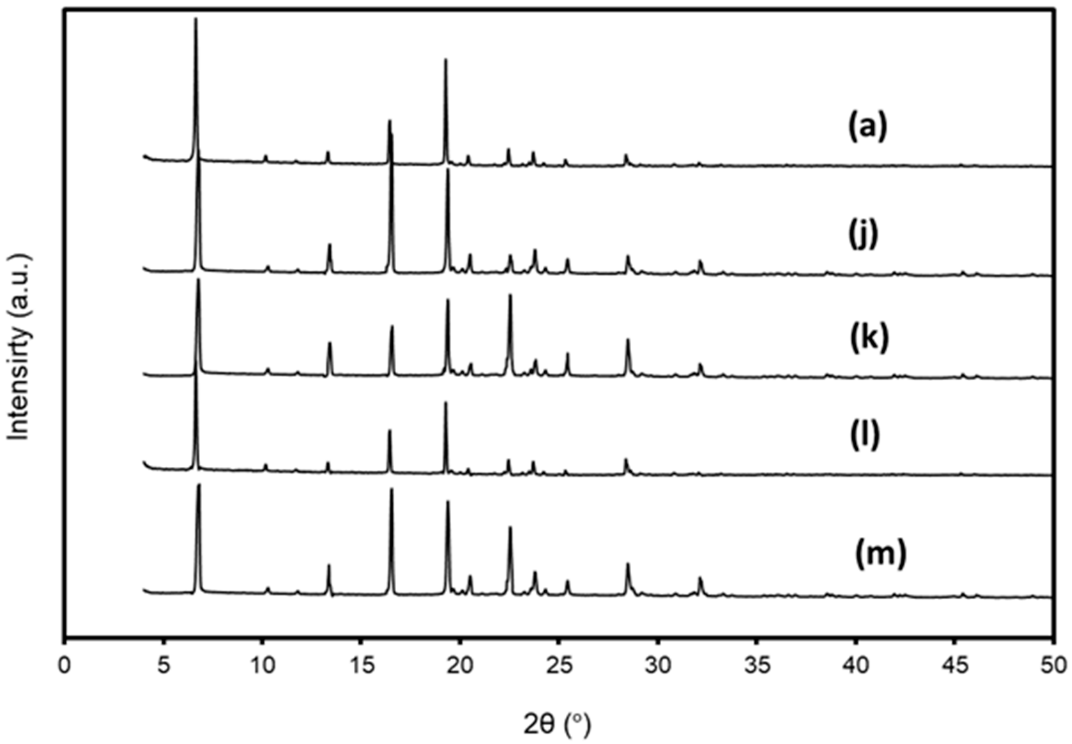

Figure S1. Powder X-ray diffraction profiles of (a) raw *trans*-resveratrol and solid phase after solubilization in (b) ethanol, (c) 1-propanol, (d) 1-butanol, (e) 1-octanol, (f) water, (g) acetone, (h) MEK, (i) MiBK, (j) acetonitrile, (k) isopropyl acetate, (l) diisopropil ether, (m) ethyl lactate.

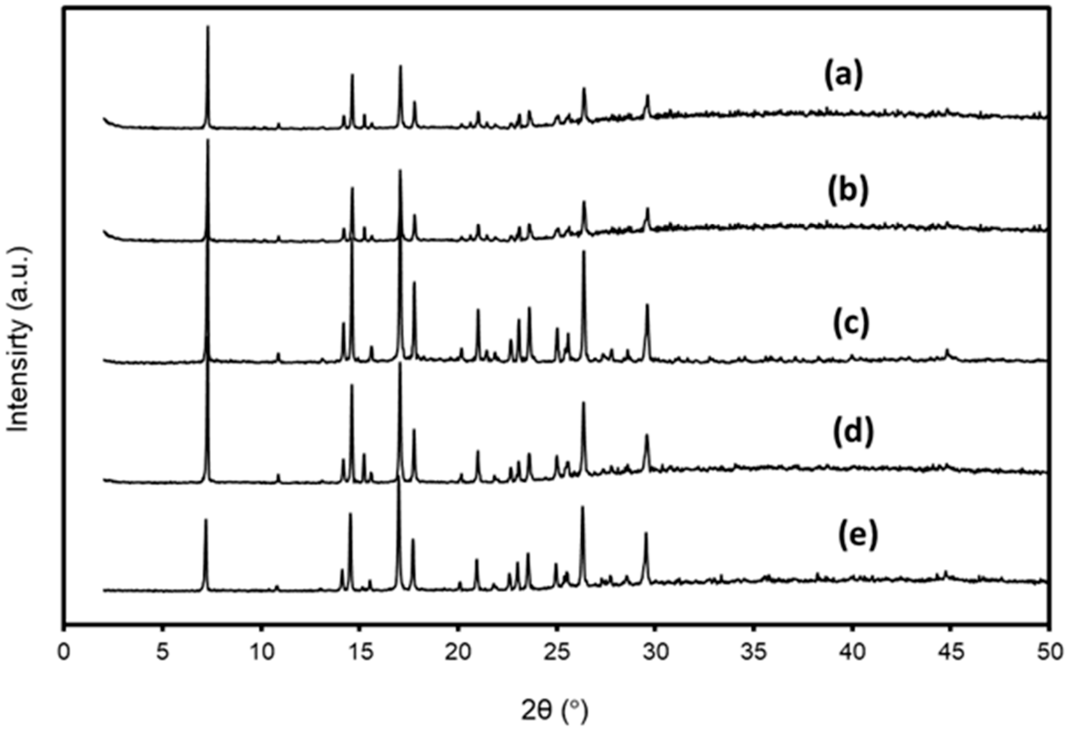

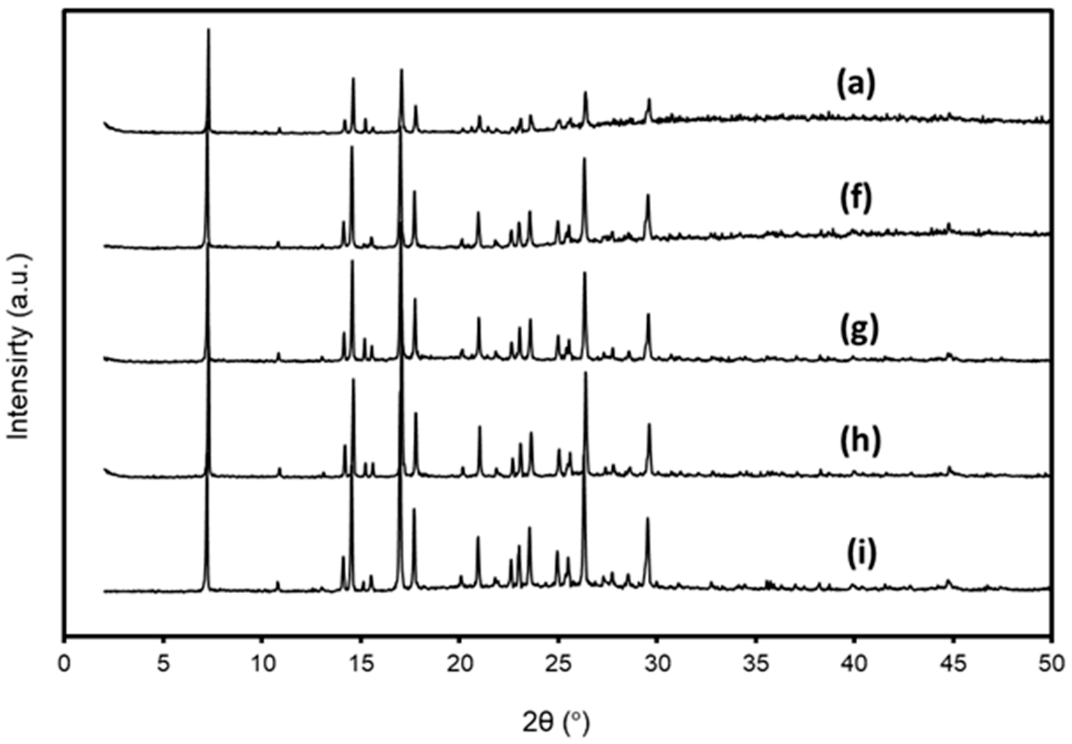
(a)
(f)
(g)
(h)
(i)
Intensirty (a.u.)
0 5 10 15 20 25 30 35 40 45 50
2θ (°)

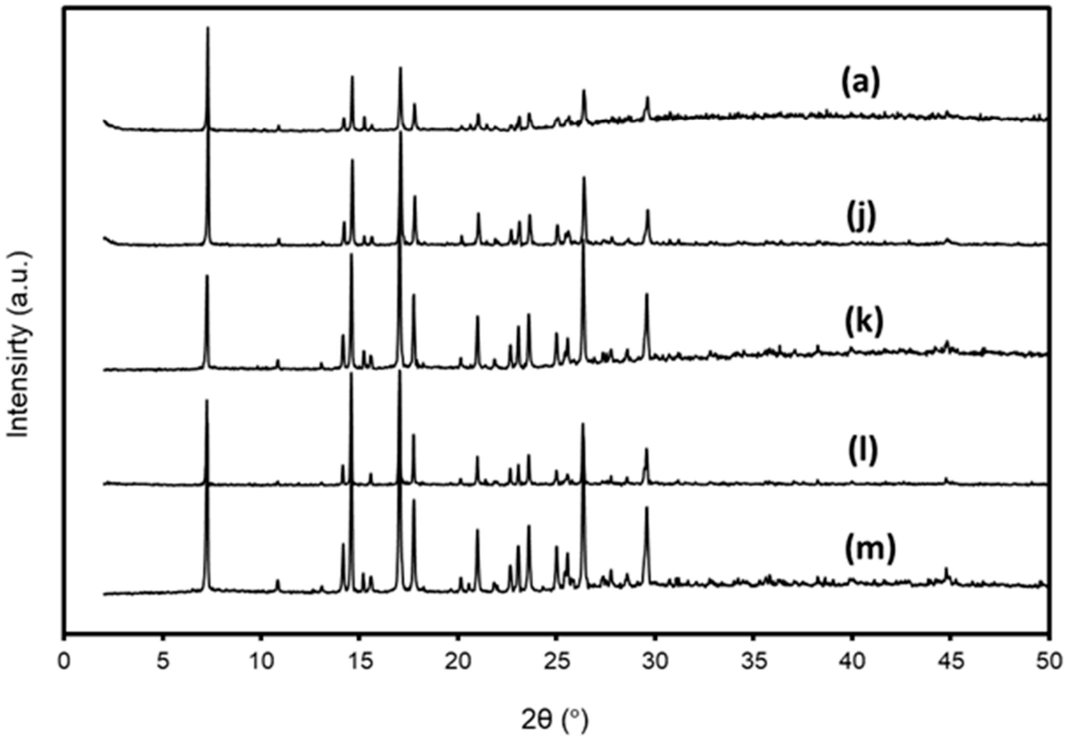
(a)
(j)
(k)
(l)
(m)
Intensirty (a.u.)
0 5 10 15 20 25 30 35 40 45 50
2θ (°)

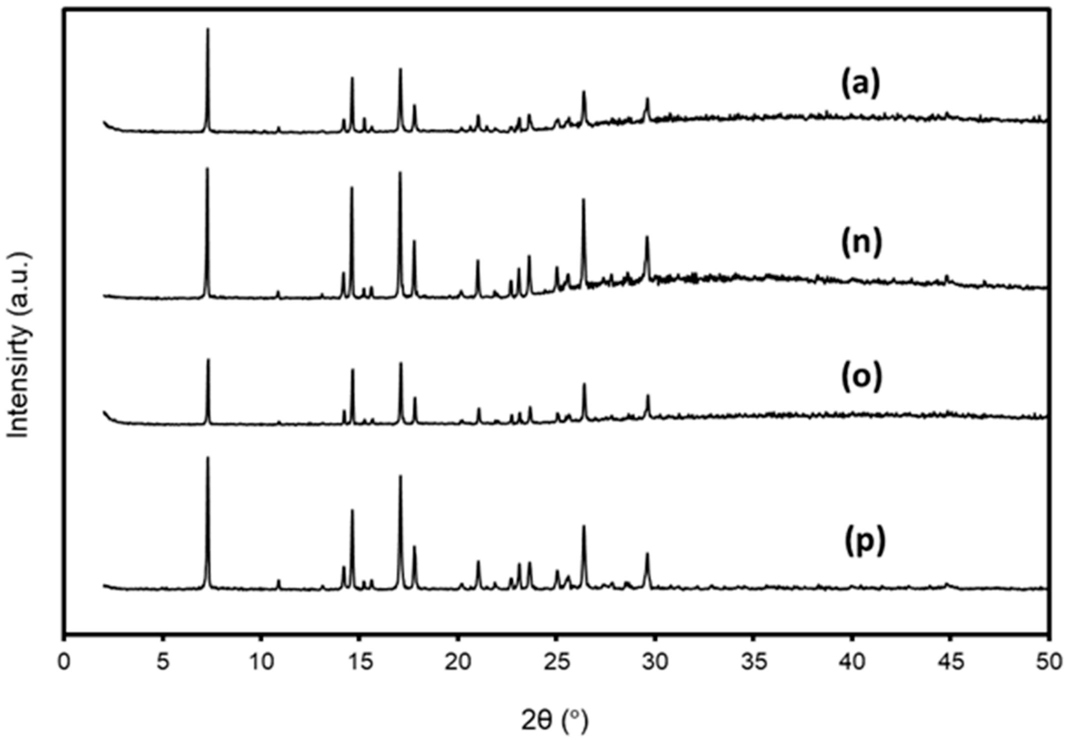

Figure S2. Powder X-ray diffraction profiles of (a) raw hesperetin and solid phase after solubilization in (b) methanol, (c) ethanol, (d) 1-propanol, (e) 1-butanol, (f) 1-octanol, (g) acetone, (h) MEK, (i) MiBK, (j) acetonitrile, (k) ethyl acetate, (l) isopropyl acetate, (m) *n*-propyl acetate, (n) *n*-butyl acetate, (o) ethyl lactate (p) diisopropyl ether.

PXRD patterns of *trans*-resveratrol and hesperetin and their solid phase after solubilization in monosolvents are displayed in Figures S1 and S2, respectively. The diffraction results for *trans*-resveratrol in methanol, ethyl acetate, *n*-propyl acetate, *n*-butyl acetate were reported and analyzed in a previous study [1].

The close match in diffraction peak positions as well as in the full width at half maximum (FWHM) for all samples strongly indicates that none of the investigated polyphenols underwent polymorphic transformations under the recrystallization conditions employed in the pure solvents. It should be noted, however, that the PXRD intensities of some peaks of *trans*-resveratrol after solubilization in 1-propanol and MEK are lower than those of the raw materials, which is likely attributable to the smaller sample mass used in those measurements.

Overall, the consistency of the diffraction patterns confirms that the crystalline structures of both polyphenols were retained after solubilization in the tested monosolvents.

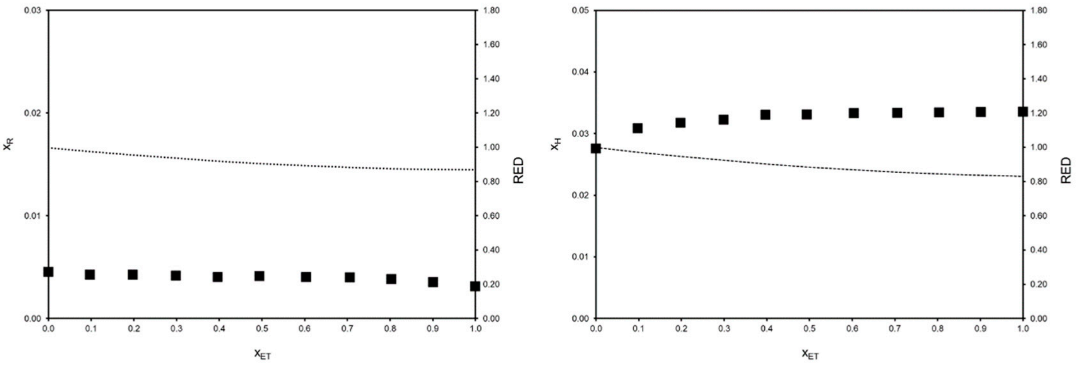

Figure S3. Mole fraction solubility of *trans*-resveratrol (x_R) and hesperetin (x_H) in methanol + ethanol (■) as a function of the mole fraction of ethanol in the binary solvent mixture (x_{ET}), at 298.15 K and 0.1 MPa. Dotted lines represent the RED value between the R_a of the binary solvent mixture with respect *p*-coumaric acid and R_0, as a function of x_{ET}.

References

[1] I. Montenegro, B. González, Á. Domínguez, and E. Gómez, "Solubility study of several polyphenolic compounds in pure and binary solvents," *J. Chem. Thermodyn.*, vol. 203, Apr. 2025, doi: 10.1016/j.jct.2024.107434.
